# Real-world outcomes in patients with relapsed and refractory multiple myeloma with prior proteasome inhibitor and lenalidomide exposure: A single-center study in Sweden

**DOI:** 10.46989/001c.146250

**Published:** 2025-12-23

**Authors:** Vincent Luong, Muhammad Kashif, Katarina Uttervall, Anna Bohlin, Annette Öster Fernström, Ying Qu, Seina Lee, João Mendes, Sandra Van Hoorenbeeck, Eva Hellqvist Franck, Jianming He, Evren Alici, Johan Lund

**Affiliations:** 1 Department of Medicine, Center for Hematology and Regenerative Medicine, Karolinska Institutet, Stockholm, Sweden; 2 Department of Hematology, Karolinska University Hospital, Stockholm, Sweden; 3 Janssen-Cilag, AB., Solna, Sweden; 4 Johnson & Johnson Innovative Medicine, Raritan, New Jersey, United States of America; 5 Johnson & Johnson, Beerse, Belgium

**Keywords:** multiple myeloma, relapsed/refractory, treatment outcomes, treatment sequence, real-world

## Abstract

The use of proteasome inhibitors (PI) and immunomodulatory drugs (IMiD) such as lenalidomide (Len) in early lines of therapy in multiple myeloma (MM) has resulted in a high proportion of patients with relapsed or refractory multiple myeloma (RRMM) being Len-refractory. Len refractoriness is associated with inferior outcomes, constituting an unmet need in clinical practice. This retrospective single-center study aimed to provide real-world characteristics and outcomes in RRMM previously exposed to PI and Len during 1-3 prior lines of therapy (LOT), stratified by Len refractoriness. RRMM patients between Jan 2017 and May 2023 were included. We studied clinical characteristics, treatments and survival outcomes. A total of 218 patients were included (n=85 Len refractory). The median progression-free survival was 13.6 months in Len-refractory versus 14.9 months in Len non-refractory patients. The median overall survival was 20.3 months in Len refractory, and not reached in Len non-refractory patients. The overall response rates to the subsequent LOT were 58.8% and 61.7% in Len refractory and non-refractory cohorts, respectively. IMiD-based and anti-CD38 monoclonal antibody-containing regimens were the most frequent subsequent LOT, 44% and 41%, respectively. This study shows suboptimal outcomes in Len refractory RRMM patients, highlighting the need to develop effective treatment options.

## Introduction

Treatment alternatives for multiple myeloma (MM) have expanded over the last two decades. The use of proteasome inhibitors (PI) and immunomodulatory drugs (IMiD) such as lenalidomide (Len) in early lines of therapy (LOT) has considerably improved patient outcomes, including prolonged survival.^1–3^ According to current European and Swedish guidelines,^4,5^ Len-containing treatment combinations, with or without PI, are recommended as front-line treatment for transplant eligible and ineligible patients, as well as for relapsed or refractory multiple myeloma (RRMM).

While most patients with MM achieve disease control and remission with PI and Len-containing treatment, many eventually become refractory to Len, or intolerant due to toxicities during prolonged treatment.^6,7^ This poses a major clinical challenge as RRMM patients refractory to Len exhibit short progression-free survival (PFS) and overall survival (OS).^8,9^ Moreover, the increasing use of Len-containing treatment regimens in front-line therapy and early-line RRMM has led to a growing number of Len refractory or intolerant patients. As Len-sparing treatment regimens have demonstrated reduced efficacy in previous studies, the optimal treatment strategy for this patient category is unclear and requires the development of effective therapeutic options.^10,11^

Recent advancements in RRMM treatment options have provided several novel alternatives for patients refractory to Len. Examples are the immunotherapies with anti-CD38 monoclonal antibodies (mAb), bispecific antibodies and CAR-T cell therapies.^12,13^ While clinical trials have proved these treatments effective for RRMM patients with prior PI and IMiD exposure, their impact in real-world clinical settings remains to be further elucidated. Thus, there is a growing need to characterize the outcomes of RRMM patients with prior lines (PL) of therapy containing PI and IMiD. Furthermore, real-world evidence from Europe, particularly the Nordic countries, on outcomes in Len refractory patients is limited. To address this, we conducted a retrospective study in a recent RRMM patient population with the objective of providing patient characteristics, treatment patterns and outcomes of early-line RRMM patients who relapsed after prior PI and Len exposure, stratified by Len refractoriness.

## Materials and Methods

### Patients

**Figure 1.**
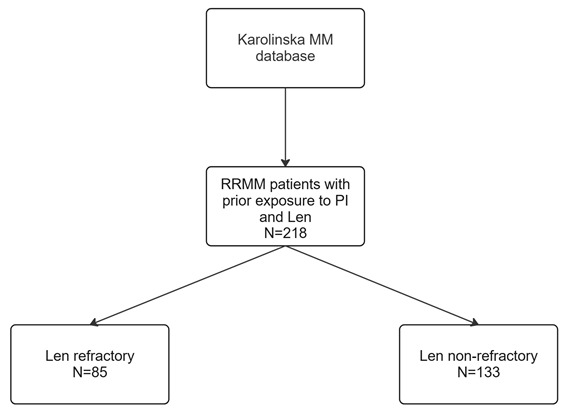
Flow chart for the patient selection process in the study. All included patients had relapsed or refractory disease at study inclusion, and started a subsequent LOT. PI included the drugs bortezomib, ixazomib and carfilzomib. Refractory status was determined according to definitions by the International Myeloma Working Group. Abbreviations: MM=multiple myeloma. PI=proteasome inhibitor. Len=lenalidomide. LOT=line of treatment. Created with Miro.com.

We conducted an observational, retrospective, single-center study with sourced data from patients with MM treated at the Department of Hematology, Karolinska University Hospital, Stockholm, Sweden. Data collection consisted of clinical and laboratory parameters, treatment patterns and survival outcomes. Patients diagnosed with RRMM after 1-3 PL, who started a subsequent LOT between January 1, 2017 and May 31, 2023 were eligible for the study. We chose this time period to reflect current clinical practice and to reduce the impact of historical treatment regimens on results. Additional inclusion criteria were: age ≥18 years at time of MM diagnosis, relapsed or refractory disease as defined by the International Myeloma Working Group (IMWG) criteria,^14^ exposure to PI and Len. A LOT was defined as ≥1 complete cycle of a single agent, a regimen consisting of a combination of several drugs, or a planned sequential therapy of various regimens, as described by Rajkumar et al.^15^ Patients were stratified based on refractoriness to Len, defined as either non-responsiveness or progressive disease during Len-treatment, or progression within 60 days following cessation of Len therapy.^14^ We did not consider the dose regimen of Len in this study. The patient selection process is depicted in **Figure 1**.

### Outcomes

The main outcomes for the study were PFS and OS, evaluated and compared between Len refractory and Len non-refractory patients. The index time point (T0) for calculation of outcomes was defined as the start of a subsequent LOT (first dose) after study inclusion. We defined PFS from T0 to the date of disease progression according to IMWG criteria or death of any cause, whichever came first. OS was defined from T0 to the date of death of any cause. Patients were censored at the date of last follow-up (visit) if no event had occurred at data cut-off. Univariate Cox regression models were employed to evaluate prognostic variables for PFS and OS. Variables with a p-value <0.05 in the univariate analyses were subsequently included in multivariate Cox models to identify independent prognostic predictors.

Additionally, we assessed the time to next treatment (TTNT) defined from T0 to the start of a subsequent LOT (first dose) or death, and time on treatment defined from T0 to last dose of the LOT or death. Patients were censored at the date of last follow-up (visit) if no event had occurred at data cut-off. Further, we assessed overall response rate (ORR) to the subsequent regimen for both groups, defined as partial response (PR) or better according to IMWG criteria.

### Treatment patterns

The treatment attrition sequence was assessed from front-line treatment until death or data cut-off date, up to the fourth LOT. The subsequent treatment regimen after study inclusion was reported separately. Treatment regimens were grouped into six categories shown in **Table 1**. We did not assess the dose of drugs, drug administration frequency and whether corticosteroids were used or not.

**Table 1.** Treatment combinations used in the study.*

*Treatment combination*	*Details*
PI combination	Bortezomib, carfilzomib or ixazomib with steroids and/or cytotoxic drugs
IMiD combination	Lenalidomide, pomalidomide or thalidomide with steroids and/or cytotoxic drugs
PI-IMiD combination	Bortezomib, carfilzomib or ixazomib and lenalidomide, pomalidomide or thalidomide with steroids and/or cytotoxic drugs
CD38 mono	Anti-CD38 mAb monotherapy
CD38 combination	Anti-CD38 mAb and one or several of PI, IMiD or cytotoxic drugs
Other	Cytotoxic drugs or other infrequently used agents

* Abbreviations: PI=proteasome inhibitor. IMiD=immunomodulatory drug. CD38=anti-CD38 monoclonal antibody.

### Statistical analysis

Descriptive data parameters were summarized with numerical counts and percentages. Medians, ranges, interquartile ranges (25/75 percentile) and two-sided 95% confidence intervals (CI) were used. Missing values were reported and not imputed in the analyses. No hypothesis testing was undertaken for survival outcomes as this study was observational in nature, and our objective was to provide descriptive outcomes. Descriptive analysis was performed in Microsoft Excel (version 2408, Microsoft) and IBM SPSS Statistics (version 29.0.1, IBM). Cox regression models and time-to-event analyses were performed using R (version 4.4.1, R Foundation for Statistical Computing). Since patients were included from 1-3 PL and stratified by Len-exposure and refractory status, no baseline adjustment was considered.

## Results

Of patients with RRMM at our center matching the study criteria, 218 were identified eligible at 1-3 PL. Overall, the median age at relapse was 72.1 years (range 36.2-93.6), with 58.3% being male, and a median duration of 2.4 (IQR 0.9-4.4) years had elapsed since MM diagnosis. Among them, 55 patients (25.3%) were refractory to PI, 88 (40.4%) to an IMiD, and 39 to both PI and IMiD (17.9%). Seventeen patients (7.8%) were previously exposed to anti-CD38 mAb (thus triple-class exposed). The proportions of 1, 2, and 3 PL were 49.1%, 46.3%, and 4.6%, respectively. Due to the low proportion of patients with 3 PL, they were combined with patients with 2 PL for the survival analyses. Most patients (71.6%) had an ECOG PS score 0 to 1 (including estimated values from charts where data was not explicitly available in records). Eighty-five patients (39.0%) were classified as Len refractory during previous LOT, while 133 (61.0%) were Len non-refractory. Clinical characteristics for Len refractory and non-refractory patients are presented in **Table 2**.

**Table 2.** Characteristics of Len refractory and Len non-refractory patients.^a^

*Characteristic*	*Len refractory* *n=85*	*Len non-refractory* *n=133*
Median age, y (range) ≥65, n (%) ≥75, n (%)	71.1 (42.3-93.6) 69 (81.2) 27 (31.8)	73.0 (36.2-92.9) 99 (74.4) 52 (39.1)
Years from MM diagnosis to study inclusion y, median (IQR 25/75)	1.8 (0.5-3.5)	2.8 (1.2-4.8)
Male gender, n (%)	48 (56.5)	79 (59.4)
ISS at MM diagnosis, n (%) I II III Unknown	15 (17.7) 34 (40.0) 23 (27.1) 13 (15.3)	18 (13.5) 65 (48.9) 26 (19.6) 24 (18.1)
Cytogenetic profile at MM diagnosis, n (%) High-risk^b^ Standard Unknown	22 (25.9) 36 (42.4) 27 (31.7)	33 (24.8) 53 (39.9) 47 (35.3)
ECOG PS, including estimated^c^, n (%) 0 1 2 3 4 Unknown	1 (1.2) 57 (67.1) 20 (23.5) 5 (5.9) 0 (0) 2 (2.4)	4 (3.0) 94 (70.7) 27 (20.3) 5 (3.8) 0 (0) 3 (2.3)
Type of MM, n (%) IgG IgA Light Chain Other	50 (58.8) 18 (21.2) 15 (17.6) 2 (1.2)	83 (62.4) 21 (15.8) 24 (18.0) 5 (3.8)
Hemoglobin g/L, median (range)	111 (68-177)	124 (68-177)
eGFR mL/min/1.73 m^2^, median (range)	61 (13-114)	63 (6-142)
Prior lines of treatment, n (%) 1 2-3	35 (41.2) 50 (58.8)	72 (54.1) 61 (45.9)
Prior anti-CD38 mAb exposure, n (%)	9 (10.6)	8 (6.0)
Prior autologous stem cell transplant, n (%)	21 (24.7)	54 (40.6)

^a^ Abbreviations: ISS=International Staging System. ECOG PS=Eastern Cooperative Oncology Group Performance Status. MM=multiple myeloma. eGFR=estimated glomerular filtration rate (LMR18 formula). mAb=monoclonal antibody.

^b^ Defined as presence of t(4;14), t(14;16), del17p or amp/gain1q.

^c^ Determined by chart review by two physicians if missing.

**Figure 2.**
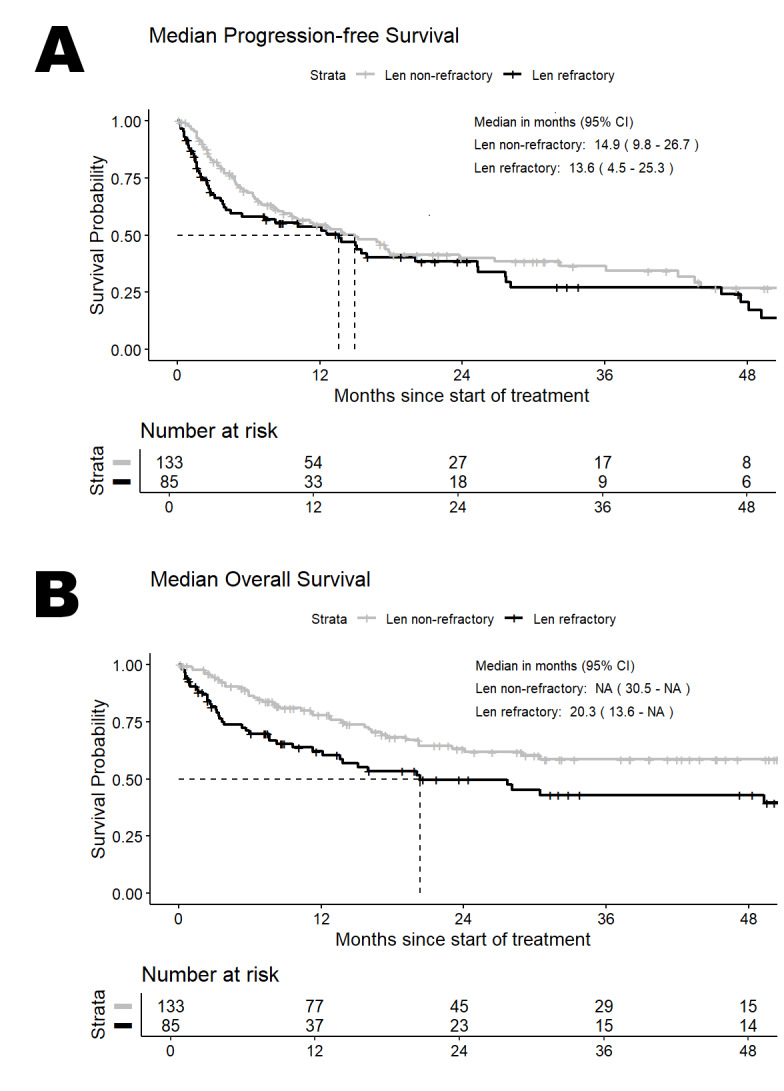
Median Progression-free Survival (A) and median Overall Survival (B) in Len refractory and Len non-refractory patients. The Kaplan-Meier method was used to estimate survival. Survival time was calculated from the start of the subsequent treatment after study inclusion. Abbreviations: Len=lenalidomide. CI=confidence interval.

During follow-up, the median PFS (mPFS) in the full patient population was 14.0 (95%CI: 10.1-17.9) months and median OS (mOS) was 51.2 (95%CI: 24.1-not evaluable, N.E.) months. In the Len refractory cohort (n=85), mPFS and mOS were 13.6 (95%CI: 4.5-25.3) months and 20.3 (95%CI: 9.6-N.E.) months, respectively. Among the Len-exposed, non-refractory patients (n=133), mPFS was 14.9 (95%CI: 9.8-26.7) months and mOS was not reached. mPFS and mOS for both cohorts are shown in **Figure 2**. Survival outcomes for both cohorts by prior line of therapy are shown in Online Resource **1**.

**Figure 3.**
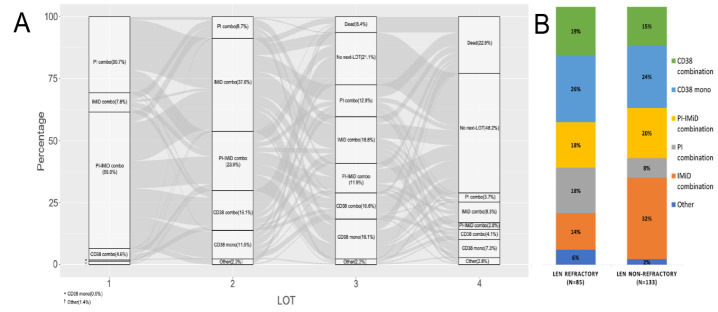
Treatment sequence from front-line (A) and most frequent subsequent treatment (B) for patients in the study (n=218). Treatment sequence shown from front-line and onwards; study entry timepoint not considered. No patient received >4 treatment regimens due to the inclusion criteria. Subsequent treatment stratified by Len refractory status. Treatment combinations are described in Table 1. Abbreviations: LOT=line of therapy. PI=proteasome inhibitor. IMiD=immunomodulatory drug. CD38 =anti-CD38 mAb.

The most common subsequent treatment combinations after study inclusion were IMiD-based regimens and anti-CD38 mAb monotherapy (25% each), followed by PI-IMiD combinations (19%) and anti-CD38 mAb combinations (17%). For Len refractory patients, anti-CD38 mAb monotherapy (26%) was the most frequent subsequent regimen, followed by anti-CD38 combinations (19%) and PI combinations or PI-IMiD combinations (18% each). Among Len non-refractory patients, the most common regimens were IMiD combinations (32%), anti-CD38 mAb monotherapy (24%) and PI-IMiD combinations (20%). The sequence from frontline treatment onwards and most common subsequent treatment is presented in **Figure 3**. This constitutes all patients included at 1, 2 and 3 PL. Additional details on subsequent treatment regimens are provided in **Online Resource 2**. We conducted separate survival analyses for PFS and OS in Len refractory and Len non-refractory patients, stratified by exposure to anti-CD38 mAb during the subsequent LOT following study inclusion. Len refractory patients exhibited a tendency toward shorter PFS and OS compared to non-refractory patients, as detailed in **Online Resource 3**.

**Figure 4.**
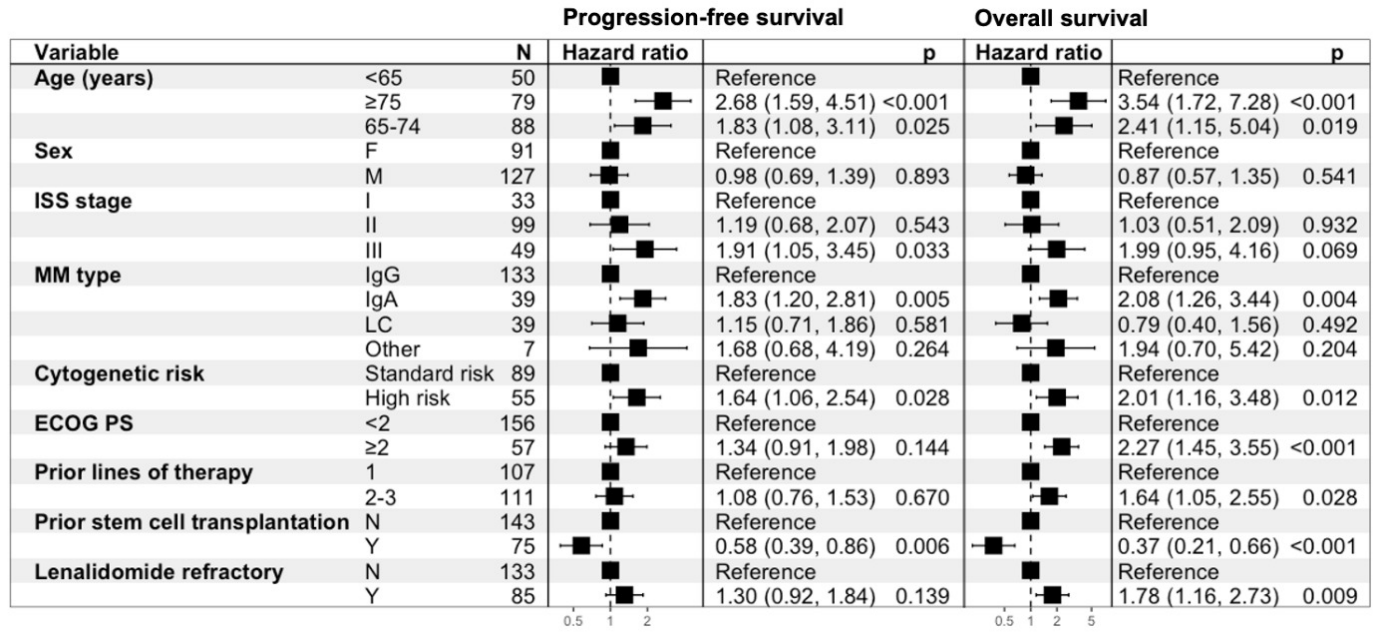
Univariate Cox regression model for Progression-free Survival and Overall Survival. Cytogenetic high risk defined as presence of t(4;14), t(14;16), del17p or amp/gain1q. Abbreviations: F=female. M=male. ISS=International Staging System. MM=multiple myeloma. LC=light chain. ECOG PS=Eastern Cooperative Oncology Group Performance Status.

Cox regression analysis indicated that age 65-74, age ≥75, high-risk cytogenetic profile, IgA MM isotype and no prior autologous stem cell transplant were associated with worse outcomes for PFS and OS (p<0.05). ISS III was prognostic for worse PFS. Variables associated with worse OS were ECOG PS ≥2 (including estimated), 2-3 PL and Len refractoriness. Prognostic variables for PFS and OS are shown in **Figure 4**. We performed a separate Cox regression analysis to evaluate the impact of subsequent treatment regimens on survival outcomes. We found that PI-IMiD and anti-CD38 mAb combinations were associated with superior PFS. For OS, only anti-CD38 mAb combinations were prognostic for improved outcomes. The effects of different subsequent LOT are summarized in **Table 3**.

**Table 3.** Univariate Cox regression analysis of Progression-free Survival and Overall Survival by subsequent treatment regimen.*

	**Progression-free survival**			**Overall Survival**		
Variable	HR	95% CI	p-⁠value	HR	95% CI	p-⁠value
PI combination	Reference			Reference		
IMiD combination	0.63	0.36-1.09	0.098	0.52	0.26-1.03	0.062
PI-IMiD combination	0.47	0.25-0.88	0.019	0.51	0.24-1.09	0.084
CD38 mono	0.83	0.48-1.42	0.499	0.74	0.38-1.42	0.363
CD38 combination	0.22	0.09-0.53	<0.001	0.34	0.13-0.88	0.027
Other	1.04	0.44-⁠2.47	0.930	1.41	0.54-⁠3.68	0.484

* Treatment combinations are described in Table 1. Abbreviations: HR=Hazard ratio. PI=proteasome inhibitor. IMiD=immunomodulatory drug. CD38=anti-CD38 monoclonal antibody.

To further evaluate the interaction between prognostic factors, we constructed multivariate models for PFS and OS, including variables with a p-value <0.05 from univariate analyses. We found that IgA MM isotype, cytogenetic high-risk and anti-CD38 mAb combination treatment were independent predictors of PFS. For OS, independent predictors were cytogenetic high-risk, ECOG PS ≥2, Len refractoriness and anti-CD38 mAb combination treatment. The multivariate model results are presented in **Table 4**.

**Table 4.** Multivariate Cox Regression models for Progression-free Survival and Overall Survival.^a^

	**Progression-free survival**			**Overall Survival**		
Variable	HR	95% CI	p-⁠value	HR	95% CI	p-⁠value
Age ≥65	1.07	0.57-⁠2.02	0.833	0.82	0.33-⁠2.01	0.661
ISS I	Reference			ns		
II	0.97	0.53-1.78	0.922	ns		
III	1.34	0.68-2.68	0.400	ns		
IgA MM type^b^	2.36	1.25-4.44	0.008	2.14	0.97-4.73	0.060
Cytogenetic high-risk^c^	1.74	1.07-2.83	0.026	2.78	1.52-5.09	<0.001
ECOG PS ≥2^d^	ns			3.05	1.56-5.98	<0.001
2-3 prior LOT	ns			1.45	0.80-2.62	0.217
Prior ASCT	0.69	0.39-1.20	0.188	0.59	0.27-1.29	0.187
Len refractory status	ns			1.93	1.03-3.60	0.040
Subsequent treatment^e^						
PI	Reference			Reference		
IMiD	0.81	0.34-1.90	0.622	0.88	0.33-2.37	0.798
PI-IMiD combo	0.79	0.32-1.95	0.611	0.94	0.33-2.63	0.901
CD38 mono	1.36	0.58-3.22	0.483	1.39	0.52-3.72	0.511
CD38 combo	0.22	0.06-0.86	0.030	0.18	0.04-0.81	0.025
Other	1.70	0.48-5.98	0.408	0.14	0.26-4.90	0.864

^a^ Variables with p <0.05 in univariate Cox regression analyses were included (135 cases and 81 events for PFS, 140 cases and 52 events for OS). Abbreviations: HR=hazard ratio. CI=confidence interval. ISS=International Staging System. MM=multiple myeloma. ECOG PS=Eastern Cooperative Oncology Group Performance Status. LOT=line of therapy. ASCT=autologous stem cell transplant. Len= Lenalidomide. PI=proteasome inhibitor. IMiD=immunomodulatory drug. CD38=anti-CD38 monoclonal antibody. ns=not significant in the univariate analysis.

^b^ IgA MM type was compared to all other isotypes.

^c^ Defined as presence of t(4;14), t(14;16), del17p or amp/gain1q.

^d^ Determined by chart review by two physicians if missing.

^e^ Treatment combinations are described in Table 1.

In our additional analysis, we determined that the median TTNT for the LOT before study inclusion was 7.6 (95%CI: 5.8-10.7) months in Len refractory and 25.6 (95%CI: 20.5-30.0) months in Len non-refractory patients. The median time on treatment for previous LOT was 6.1 (95%CI: 4.8-8.4) months in Len refractory and 6.0 (5.2-7.0) months in Len non-refractory patients. For the subsequent LOT following study inclusion, median TTNT was 12.1 (95%CI: 5.5-25.4) months in Len refractory and in Len non-refractory patients 15.1 (95%CI: 10.7-29.0) months. Median time on treatment for the subsequent LOT was 7.6 (95%CI: 3.3-14.9) months for Len refractory and 7.3 (95%CI: 5.5-13.8) months for Len non-refractory patients. ORR to the subsequent regimen was 58.8% among Len refractory and 61.7% among Len non-refractory patients. Thirty-six and a half % of Len refractory and 42.1% of Len non-refractory patients achieved VGPR or better response. ORR by Len refractory status and response categories is presented in **Table 5**.

**Table 5.** Overall Response Rate (ORR) to subsequent line of treatment for Len refractory and Len non-refractory patients by category.^a^

*Response*	*Len refractory* *n=85*	*Len non-refractory* *n=133*
ORR % (95%CI)	58.8 (48.2-68.7)	61.7 (53.2-69.5)
By Category, n (%)		
CR^b^	10 (11.8)	11 (8.3)
VGPR	21 (24.7)	45 (33.8)
PR	19 (22.4)	26 (19.6)
SD	21 (24.7)	44 (33.1)
PD	6 (7.1)	3 (2.3)
Not evaluable	8 (9.4)	4 (3.0)

^a^ Abbreviations: Len=lenalidomide, ORR=overall response rate, CR=complete response, VGPR=very good partial response, PR=partial response, SD=stabile disease, PD=progressive disease.

^b^ Bone marrow assessment not routinely performed for all patients during follow-up at our clinic, resulting in low number of CR patients (mainly investigated in clinical trial settings or after autologous stem cell transplant).

## Discussion

This is the first study in Sweden reporting the treatment pattern and outcomes of a difficult-to-treat RRMM cohort, with prior PI and Len exposure. Overall, we show that PFS of early-line RRMM patients (1-3 PL) with previous Len-exposure is short. Patients refractory to Len have particularly unfavorable outcomes with reduced OS, highlighting the substantial unmet clinical need for more efficacious and safe therapeutic options.

Our main aim was to explore outcomes in Len refractory patients, who had a mPFS of 13.6 months, mOS of 20.3 months and ORR of 58.8% after the subsequent LOT, representing the most recent clinical data on Swedish RRMM patients, findings which are in line with previous European real-world reports on Len refractory, early-line RRMM patient cohorts.^16,17^ A single-center retrospective study in Greece^16^ included 249 Len-exposed patients following front-line treatment, where 55.4% were Len refractory, had an ORR of 53% to the subsequent LOT, and estimated mOS of 23.8 months and mPFS of 10.7 months. A retrospective study performed at two institutions in the UK,^17^ on 198 RRMM patients exposed to Len with a median of 2 PL showed a mOS of 14.7 months after becoming refractory to Len or cessation of the drug for other reasons. The slightly improved survival outcomes observed in our study may be attributed to the comparably higher usage of anti-CD38 mAb-based treatments following Len exposure, which for all anti-CD38 mAb-containing regimens were 42% of subsequent treatments for the full cohort. However, comparisons between these studies and ours should be interpreted with caution, given the limitations of real-world evidence with single- or double-center study designs, heterogeneity in patient cohorts, difference in number of prior LOT, variations in treatment guidelines and differing study periods.

Further, a USA study reported real-world outcomes in RRMM at 1-3 PL exposed to PI and refractory to Len, and found a mPFS of 6.5 (95%CI: 5.6-7.0) months and a mOS of 44.4 (95%CI: 40.1-51.4) months.^18^ The authors utilized the Flatiron Health database, including patients between January 2016 and April 2022, (n=1455). Of note, patients in the that study had ECOG 0-1 and their median age was 69 years. Similarly, a USA-based study among patients ≥65 years (n=1297) in the SEER-Medicare database showed a mOS of 29.3 (95%CI, 25.1-33.9) months and a median TTNT of 8.5 (95%CI: 7.7-9.7) months.^19^ Patients were included between 2016 and 2020, had 1-3 PL with prior PI and IMiD exposure, and were Len refractory. The median age of patients was 75 years and the authors reported that anti-CD38 mAb was commonly used in subsequent treatment (43.7%). When comparing these studies with ours, it is important to consider possible differences in healthcare utilization and in drug reimbursement policies in Swedish/European settings and the USA, as well as possible differences in patient cohorts, such as age and ECOG PS, among other factors.

Large randomized clinical trials have provided similar findings to ours in the early-line (1-3 PL) Len exposed RRMM setting. Our review identified two studies:

A. The OPTIMISMM trial,^20^ enrolled 559 Len exposed RRMM patients with 1-3 PL, with 70% classed as Len refractory. Patients were randomized to pomalidomide-bortezomib-dexametasone (PVd) or bortezomib-dexametasone (Vd) treatment. The former group had superior mPFS (11.20 versus 7.10 months, HR 0.61, p<0.0001), with an ORR of 82.2% versus 50.0%. Among Len refractory patients, the mPFS was improved in the PVd arm (9.53 versus 5.59 months, HR 0.65, p=0.0008). Of note, most patients (94%) in that study had an ECOG PS 0-1, median age <70 years, 51% had ISS stage I, approximately 20% had cytogenetic high-risk disease and 58% had a prior autologous stem cell transplant.

B. The CARTITUDE-4 trial,^21^ enrolled 419 patients previously treated with PI and IMiD, and defined as refractory to Len during 1-3 PL. Patients were randomized to standard-of-care (PVd) or daratumumab-pomalidomide-dexametasone (DPd) versus the CAR-T cell therapy ciltacabtagene autoleucel. After 15.9 months follow-up, the mPFS was not reached in the latter arm versus 11.8 months in the standard-of-care group (HR 0.26, p<0.001), with an ORR of 84.6% versus 67.3%. Notable characteristics in the study population are: median age of approximately 61 years, majority ECOG PS 0-1 (99.6%), 64% were ISS stage I, 61% had a cytogenetic high-risk disease and approximately 26% had previous anti-CD38 mAb exposure.

The mPFS in our Len refractory, real-world cohort is similar to the PVd arm in the OPTIMISMM trial and the standard-of-care arm in CARTITUDE-4. However, it is crucial to note that the median age and ISS stages are higher in our data, while fewer patients had a prior autologous stem cell transplant (compared to OPTIMISMM, not reported in CARTITUDE-4). In addition, the OPTIMISMM trial did not evaluate patients with prior or ongoing anti-CD38 mAb treatment, and more patients in CARTITUDE-4 had prior anti-CD38 mAb exposure than in our study. While our results provide additional evidence of the unmet need in Len refractory patients in real-world settings, and further corroborate the performance of the standard-of-care cohorts from the referenced clinical trials, it is crucial to consider the limitations of comparing real-world outcomes with clinical trial results.

The use of Len-free treatment regimens in RRMM following Len-exposure and especially Len refractoriness is emphasized in current European treatment guidelines.^4^ Due to the heterogeneity of prior treatment regimens, drug availability and regional cost reimbursement policies, a multitude of options are proposed without a clear recommendation of a single regimen. This is amplified by multiple novel immunotherapeutic drugs that have entered or are becoming part of the treatment paradigm in RRMM, such as anti-CD38 mAb, bispecific antibodies and CAR-T cell therapies.^12,13,22^ Even within a single country such as Sweden, the 2024 national guidelines offer many alternatives depending on prior drug exposure and eligibility to enroll in clinical trials.^5^ This demonstrates the complexity of modern RRMM treatment and the continued need to provide further evidence of outcomes in this patient population. During the study period, bispecific antibodies and CAR-T cell therapies were not available at our center outside of clinical trials. As of mid-2025, both treatment modalities have become available for routine clinical use in Sweden. We anticipate that the emerging use of these therapies will lead to improved outcomes at our center and others across the country.

In our study, TTNT for the LOT before study inclusion was markedly shorter in Len refractory patients, while time on treatment was similar in both Len refractory and non-refractory patients. This may reflect a more aggressive disease biology in Len refractory patients. Further, re-exposure to IMiD-treatment was frequent in Len non-refractory patients with 52% receiving either IMiD-based regimens or PI-IMiD combinations. In contrast, only 32% of Len refractory patients were re-treated with these regimens, in most cases with another agent from the class (pomalidomide or thalidomide). PI refractoriness was present in only 25% of all patients in our study. This likely reflects that most patients were included after relapse, without ongoing therapy, either due to discontinuation following adequate remission or due to adverse effects. Additionally, Len was frequently used as a single-agent maintenance therapy following remission or autologous stem cell transplantation.

The use of anti-CD38 mAb-based therapy was high in both cohorts (either as monotherapy or part of a combination treatment), which was administered in 45% of Len refractory and 39% of Len non-refractory patients. However, it is important to note that anti-CD38 mAb combinations included re-exposure to IMiD in our treatment groupings. In addition, our data showed a relatively low proportion of RRMM patients with prior anti-CD38 mAb exposure (7.8%). This, along with the inclusion criterion of prior PI and Len exposure reflects the limited availability of anti-CD38 mAb in front-line or second-line treatment during the study period. Anti-CD38 mAb therapy was introduced at our center in 2018, initially reserved for late-line, heavily pretreated RRMM. Its use gradually shifted toward earlier LOT and became available in frontline regimens by late 2020. However, as this study focused on patients with 1-3 PL, the increasing use of anti-CD38 mAb in early LOT is not fully captured, since most patients starting such regimens have not yet progressed by the end of the study period.

In our multivariate models, based on prognostic factors identified in the univariate Cox regression analyses, we found that cytogenetic high risk and treatment with anti-CD38 mAb combinations were independent predictors of both PFS and OS. Moreover, IgA MM isotype was an independent predictor of PFS, while ECOG PS ≥2 and Len refractory status were predictors of OS. These findings further support the observation that Len refractory patients have inferior long-term outcomes, and underscore the efficacy of anti-CD38 mAb-based combination therapies. However, these results should be interpreted with caution due to the limited cohort size, the small number of events, and the heterogeneity in treatment regimens in the study population.

Additionally, our findings are limited by the retrospective single-center study design, which limited the feasibility of further hypothesis testing for statistical purposes, which would lack power to elucidate possible differences. Further, while our study population is similar to previous real-world studies, the small number of Len refractory patients (n=85) included at 1-3 PL, results in reduced generalizability of our findings. Notably, Len non-refractory patients were younger, had more prior ASCT, and had a longer median follow-up since MM diagnosis before study inclusion. While some selection bias may be present, these parameters may also reflect the more aggressive disease biology in Len refractory patients. Additionally, our dataset did not include information on individual drug dosages, limiting the ability to assess whether treatment outcomes varied based on the dose of Len or other drugs. While this study was performed in a modern RRMM cohort with relapses between 2017 and May 2023, the rapidly evolving treatment panorama during this period may have impacted results. For instance, the use of anti-CD38 mAb, the PI carfilzomib and the IMiD pomalidomide increased at our center during the duration of the study. In particular, anti-CD38 mAb has become a key component of front-line treatment regimens for newly diagnosed MM in recent years.

In conclusion, our study of real-world outcomes of RRMM patients previously exposed to PI and Len demonstrates poor outcomes in Len refractory patients. These findings provide valuable insight into this difficult-to-treat patient category, and further validate the previous evidence from similar real-world studies and clinical trials. Although many novel treatment options are becoming available for this patient population, further studies are needed to appropriately sequence treatments and to select suitable patients.

### Authors’ Contribution

Conceptualization: Jianming He, Evren Alici, Johan Lund; Data curation: Vincent Luong, Anna Bohlin, Annette Öster Fernström; Methodology: Vincent Luong, Muhammad Kashif, Ying Qu, João Mendes, Jianming He, Evren Alici, Johan Lund; Formal analysis: Vincent Luong, Muhammad Kashif; Validation: Seina Lee, João Mendes, Sandra Van Hoorenbeeck, Eva Hellqvist Franck; Writing - original draft preparation: Vincent Luong, Katarina Uttervall, Ying Qu, Jianming He, Evren Alici, Johan Lund; Writing - review and editing: All authors.

### Competiting Interests – COPE

Katarina Uttervall: Advisory board for Johnson&Johnson, Pfizer and Sanofi; lecture fees from Johnson&Johnson. Johan Lund: Research funding from Sanofi; collaborative research funding from Pfizer and Janssen; honoraria from Amgen; advisory board for Amgen, Bristol Myers Squibb, and Sanofi. Evren Alici: Consultancy for Sanofi, Artiva, Avectas and Janssen; ownership in Vycellix. Ying Qu, Seina Lee, João Mendes, Sandra Van Hoorenbeeck, Eva Hellqvist Franck and Jianming He are employees of Johnson&Johnson Innovative Medicine. The remaining authors have no competing interests to disclose.

### Statement of Ethics

This study was approved by the Swedish Ethical Review Authority (ethical permit 2014/526-31/3 and 2019-04973) and was conducted according to the Declaration of Helsinki and the Guidelines for Good Clinical Practice. According to ethical approvals, separate informed consent was not required, as our database contains only retrospective pseudonymized data.

### Informed Consent Statement

All authors and institutions have confirmed this manuscript for publication.

## Supplementary Material

Online Resource 1 Progression-free Survival (A) and Overall Survival (B) for Len refractory and Len non-refractory patients stratified by 1 and 2-3 prior lines of treatment.*

Online resource 2 Subsequent treatment regimens administered after study inclusion, shown by treatment grouping and by number of drug classes, respectively (n=218).

Online Resource 3 Progression-free Survival (A) and Overall Survival (B) for Len refractory and Len non-refractory patients stratified by anti-CD38 mAb exposure during subsequent treatment.*

## Data Availability

Relevant data at an aggregated level are included in the paper and its Supporting Information files. Individual patient data from this study are not publicly available due to Swedish and European laws and regulations.
