# Supplementary material for: Real-world outcomes in patients with relapsed and refractory multiple myeloma with prior proteasome inhibitor and lenalidomide exposure: A single-center study in Sweden
Source: Clin Hematol Int. 2025 Dec 23;7(4):64–75. doi: 10.46989/001c.146250 (PMC12739867; doi:10.46989/001c.146250)
Supplement: Online Resource 1 — Progression-free Survival (A) and Overall Survival (B) for Len refractory and Len non-refractory patients stratified by 1 and 2-3 prior lines of treatment.* [file chi_2025_7_4_146250_323259.pdf]

Online Resource 1. Progression-free Survival (A) and Overall Survival (B) for Len refractory and Len non-refractory patients stratified by 1 and 2-3 prior lines of treatment.*

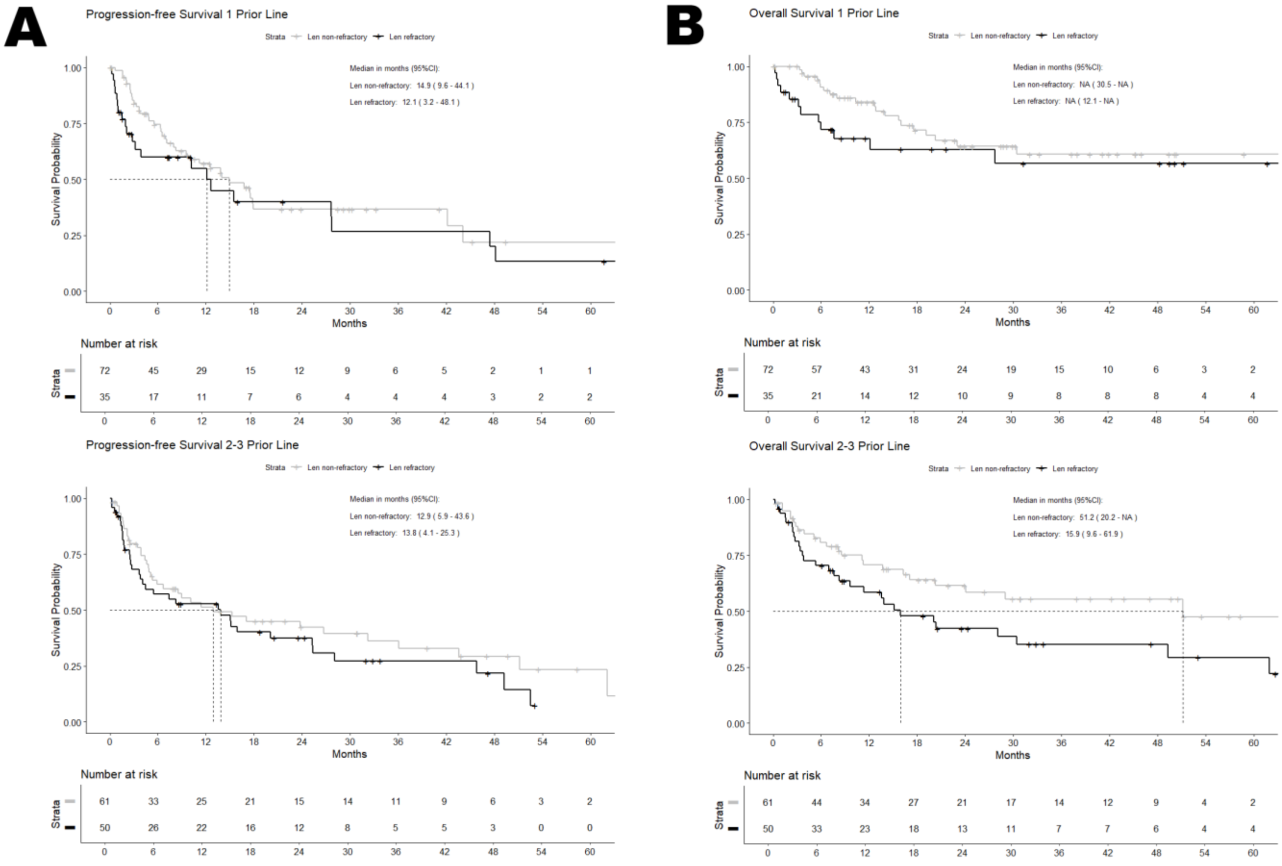

* The Kaplan-Meier method was used to estimate survival. Survival time was calculated from the start of the subsequent treatment after study inclusion. Abbreviations: Len=lenalidomide. CI=confidence interval.
