# Supplementary material for: Real-world outcomes in patients with relapsed and refractory multiple myeloma with prior proteasome inhibitor and lenalidomide exposure: A single-center study in Sweden
Source: Clin Hematol Int. 2025 Dec 23;7(4):64–75. doi: 10.46989/001c.146250 (PMC12739867; doi:10.46989/001c.146250)
Supplement: Online resource 2 — Subsequent treatment regimens administered after study inclusion, shown by treatment grouping and by number of drug classes, respectively (n=218). [file chi_2025_7_4_146250_323260.pdf]

Online Resource 2. Subsequent treatment regimens administered after study inclusion, shown by treatment grouping and by number of drug classes, respectively (n=218).

Treatment regimen by group[1], n (%)	Overall (n=218)	Len refractory (n=85)	Len non-refractory (n=133)
CD38 monotherapy	54 (24.8)	22 (25.9)	32 (24.1)
IMiD combination	54 (24.8)	12 (14.1)	42 (31.6)
PI-IMiD combination	41 (18.8)	15 (17.6)	26 (19.5)
CD38 combination	36 (16.5)	16 (18.8)	20 (15.0)
PI combination	25 (11.5)	15 (17.6)	10 (7.5)
Other	8 (3.7)	5 (5.9)	3 (2.3)
**Treatment regimen by number of drug classes[2], n (%)**			
Doublet	133 (61.0)	49 (57.6)	84 (63.2)
Triplet	72 (33.0)	29 (34.1)	43 (32.3)
Quadruplet	13 (6.0)	7 (8.2)	6 (4.5)
**Treatment regimen by individual regimen - if received by >5 patients[3], n (%)**			
Daratumumab	54 (24.8)	x	x
Lenalidomide	26 (11.9)	x	x
Pomalidomide	25 (11.5)	x	x
Carfilzomib-pomalidomide	16 (7.3)	x	x
Carfilzomib	14 (6.4)	x	x
Carfilzomib-lenalidomide	12 (5.5)	x	x
Daratumumab-lenalidomide	7 (3.2)	x	x
Daratumumab-pomalidomide	7 (3.2)	x	x
Daratumumab-bortezomib-lenalidomide	5 (2.3)	x	x
Daratumumab-carfilzomib	5 (2.3)	x	x
Bortezomib-lenalidomide	5 (2.3)	x	x
Cyclophosphamide	5 (2.3)	x	x

[1] Treatment combinations are described in Table 1.
[2] Corticosteroid use was assumed and included in calculation of number of drugs, e.g. lenalidomide with or without dexamethasone was counted as a doublet.
[3] Numbers not shown for Len refractory and non-refractory due to few patients (n<5) for most individual regimens. Regimens shown without corticosteroids.
