# Supplementary material for: Real-world outcomes in patients with relapsed and refractory multiple myeloma with prior proteasome inhibitor and lenalidomide exposure: A single-center study in Sweden
Source: Clin Hematol Int. 2025 Dec 23;7(4):64–75. doi: 10.46989/001c.146250 (PMC12739867; doi:10.46989/001c.146250)
Supplement: Online Resource 3 — Progression-free Survival (A) and Overall Survival (B) for Len refractory and Len non-refractory patients stratified by anti-CD38 mAb exposure during subsequent treatment.* [file chi_2025_7_4_146250_323261.pdf]

Online Resource 3. Progression-free Survival (A) and Overall Survival (B) for Len refractory and Len non-refractory patients stratified by anti-CD38 mAb exposure during subsequent treatment.*

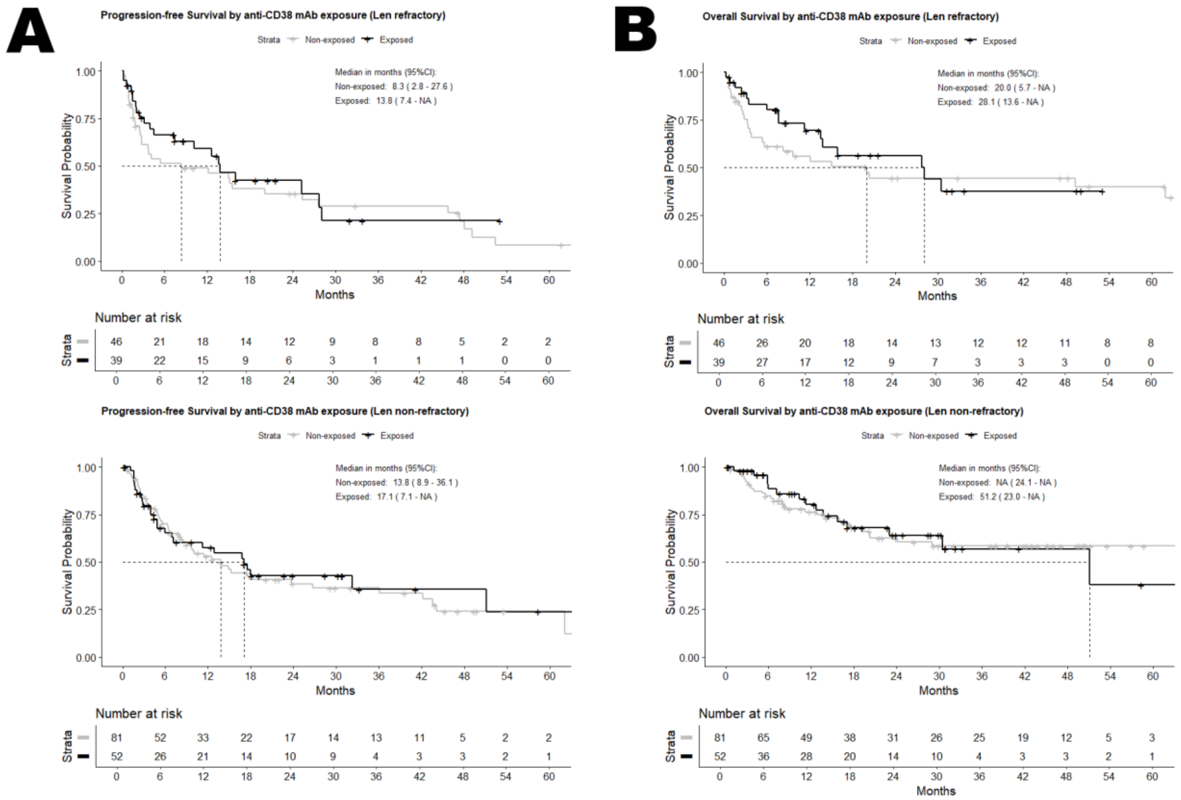

* The Kaplan-Meier method was used to estimate survival. Survival time was calculated from the start of the subsequent treatment after study inclusion. For the purpose of OS analysis, patients may have received additional LOT (up to the fourth line) following the subsequent line started at study inclusion. Len refractory patients had tendency toward inferior PFS and OS when compared to non-refractory patients, however it is crucial to consider the limited cohort sizes and the highly heterogenous anti-CD38 mAb containing regimens. Abbreviations: Len=lenalidomide. mAb=monoclonal antibody. LOT=line of therapy. CI=confidence interval.
